# Telephone-Based Health Coaching Focused on Healthy Eating in Pregnancy and Maternal Weight Outcomes

**DOI:** 10.1097/og9.0000000000000119

**Published:** 2025-10-09

**Authors:** Sylvia E. Badon, Alex Asera, Wendy T. Dyer, Huyun Dong, Charles P. Quesenberry, Elizabeth Ryan, Ashley A. Mevi, Mibhali Bhalala, Anne Srisuro, Monique M. Hedderson

**Affiliations:** Division of Research, Regional Women's Health, and the Permanente Medical Group, Kaiser Permanente Northern California, Pleasanton, California.

## Abstract

**OBJECTIVE:**

To assess whether participation in a health system–administered, telephone-based health coaching program during pregnancy was associated with lower gestational weight gain or postpartum weight retention and whether associations differed by the number of sessions or timing of participation.

**METHODS:**

In this retrospective cohort study, we used electronic health record data to identify pregnant Kaiser Permanente Northern California members with referral to a telephone-based health coaching program focused on healthy eating during pregnancy (Wellness Coaching in Pregnancy) from 2020 to 2022. Outcomes of interest were total gestational weight gain and postpartum weight retention up to 14 months postpartum. We also assessed the number and trimester of completed sessions. Regression models, weighted by the inverse probability of treatment weights calculated using a propensity score model containing maternal demographic, behavioral, and health characteristics, were used to estimate associations of Wellness Coaching in Pregnancy participation with gestational weight gain categories based on Institute of Medicine (IOM, now known as the National Academy of Medicine) recommendations and continuous postpartum weight retention. We also examined results stratified by the number of sessions completed and by trimester of initiation of Wellness Coaching in Pregnancy.

**RESULTS:**

The final cohort consisted of 931 individuals with 935 pregnancies (four individuals had two pregnancies during the study period); 525 pregnant individuals (56.1%) participated in Wellness Coaching in Pregnancy after referral, with a median of two completed sessions. Overall, Wellness Coaching in Pregnancy was not associated with gestational weight gain or postpartum weight retention. In secondary analyses, completing the first session of Wellness Coaching in Pregnancy in the first trimester was associated with 40% lower odds of gestational weight gain above IOM recommendations (adjusted odds ratio 0.60; 95% CI, 0.39–0.94) and 2.2 kg lower adjusted mean difference in postpartum weight retention up to 14 months postpartum (95% CI, −3.7 to −0.7) compared with individuals who were referred but did not participate in Wellness Coaching in Pregnancy. The number of sessions completed was not associated with gestational weight gain or postpartum weight retention.

**CONCLUSION:**

Within a large, integrated health care delivery system, an individualized health coaching program focused on healthy eating during pregnancy was not associated with gestational weight gain and postpartum weight retention overall. However, when health coaching was initiated in early pregnancy, it was beneficially associated with reduced excessive gestational weight gain and postpartum weight retention.

## Abstract 2

Early pregnancy initiation of an individualized health coaching program focused on healthy eating was associated with reduced high gestational weight gain and postpartum weight retention.

In North America, nearly half of pregnant individuals exceed Institute of Medicine (IOM, now known as the National Academy of Medicine) gestational weight gain recommendations.^1–3^ Excessive gestational weight gain is associated with increased risk of pregnancy complications,^4–6^ as well as postpartum weight retention, which increases long-term risk for obesity, type 2 diabetes, and cardiovascular disease.^7–12^ The American College of Obstetricians & Gynecologists’ Committee Opinion No. 548, “Weight Gain During Pregnancy,” emphasizes the need to reduce rates of excessive gestational weight gain to improve maternal and child health.^1^

Meta-analyses support the effectiveness of dietary interventions for reducing excessive gestational weight gain,^13,14^ but most have been delivered in person, posing access barriers. Telephone-based interventions can improve reach and convenience,^15^ yet prior studies of telephone-based health coaching focused on diet in pregnancy show mixed results.^15–18^ Most used research staff to deliver coaching, limiting generalizability to health care delivery settings. Only two studies, both in Australia, evaluated telephone-based health coaching embedded in health systems; neither found associations with gestational weight gain, though one showed promising results for participants who completed all sessions.^19,20^

The U.S. Preventive Services Task Force recommends that clinicians offer behavioral counseling interventions during pregnancy to promote healthy gestational weight gain.^21,22^ Given this recommendation, there is a need to identify effective interventions for healthy weight gain within clinical settings. We assessed whether participation in a health system–administered, telephone-based health coaching program during pregnancy was associated with gestational weight gain or postpartum weight retention, and whether associations varied by number of sessions completed or timing of program initiation. We hypothesized that participation in a health system–administered telephone-based health coaching program during pregnancy would be associated with lower risk of excessive gestational weight gain and lower postpartum weight retention, with a dose-response relationship between number of sessions completed and stronger associations for program initiation in the first and second trimesters.

## METHODS

We used electronic health record (EHR) data from Kaiser Permanente Northern California, an integrated health care delivery system that provides care for more than 60,000 pregnant individuals annually in Northern California, to conduct a retrospective cohort study. Kaiser Permanente Northern California membership represents the diverse population of the Northern California region in terms of demographic, ethnic, and socioeconomic characteristics.^23^ This study was determined to be exempt by the Kaiser Permanente Northern California IRB, and use of birth certificate data was approved by the California Committee for the Protection of Human Subjects. The requirement for informed consent was waived.

Kaiser Permanente Northern California EHRs were used to identify deliveries between January 2020 and December 2022 with a referral to the Wellness Coaching in Pregnancy program at any time during pregnancy. Clinicians referred pregnant individuals to Wellness Coaching in Pregnancy based on their judgment of who may benefit from the program and patient interest in the program. Individuals with nonsingleton pregnancies, those with pregnancy outcomes at less than 20 weeks after the last menstrual period, those with a gap in Kaiser Permanente Northern California membership, and those with pregnancies that ended in nonlive births were excluded from the cohort. The final cohort consisted of 935 pregnancies in 931 individuals (four individuals had two pregnancies during the study period).

The Kaiser Permanente Northern California Wellness Coaching in Pregnancy program is a one-on-one telephone-based health coaching program available at no cost to Kaiser Permanente Northern California members. In 2020, the Wellness Coaching in Pregnancy program was expanded to offer health coaching focused on healthy eating during pregnancy, with the goal of helping more pregnant members achieve healthy gestational weight gain. Wellness Coaching in Pregnancy is a motivational, interviewing-based program, and wellness coaches are master's-level clinical health educators trained in motivational interviewing strategies. In addition, wellness coaches were trained on how to tailor healthy eating conversations to pregnant members, including gestational weight gain recommendations, pregnancy-specific benefits of eating fish and shellfish, foods to avoid during pregnancy, and discouraging dieting as well as “eating for two.” Wellness coaches also received additional education resources specific to healthy eating and pregnancy that could be shared with members.

The number, frequency (maximum six sessions in a 6-month period), and spacing of sessions was decided on between the patient and their wellness coach based on patient goals, preferences, and schedule. Sessions were 15 to 20 minutes long. We identified completed Wellness Coaching in Pregnancy sessions in the EHR. We categorized participation in Wellness Coaching in Pregnancy as a binary variable (any participation vs no participation), by the total number of sessions completed (zero, one, two, or three or more sessions) to explore a potential dose–response relationship, and by trimester of first completed Wellness Coaching in Pregnancy session (first, second, third) to explore the effect of timing of initiation of Wellness Coaching in Pregnancy.

We calculated total gestational weight gain as the difference between weight measured closest to, but before, delivery and prepregnancy weight (closest clinically measured weight to pregnancy onset within 365 days before pregnancy onset to within 70 days after pregnancy onset). We used the gestational weight gain adequacy ratio (observed/expected gestational weight gain at gestational week of delivery) to categorize gestational weight gain according to IOM recommendations (below, within, or above recommendations, Appendix 1 available online at http://links.lww.com/AOG/E358).^2^ Expected gestational weight gain was calculated using the IOM recommended first-trimester weight gain and the recommended rate of weight gain in the second and third trimesters, with the following formula: expected gestational weight gain=recommended first-trimester weight gain+(gestational age at delivery−13)×recommended rate of weight gain in the second and third trimesters. Using the gestational weight gain adequacy ratio rather than total gestational weight gain to create IOM gestational weight gain categories allowed us to include preterm deliveries (8.6%) for which IOM total gestational weight gain categories may not be appropriate.

All clinically measured weights between 31 and 425 days after delivery were extracted from the EHR, and one weight per month was retained. For individuals with multiple weights in the same postpartum month, we selected the first weight in that month. For each participant, we calculated postpartum weight retention at each postpartum month with weight data available by subtracting prepregnancy weight from postpartum weight. Monthly postpartum weight retention data were used as outcomes in repeated measures models.

Information about maternal age, maternal smoking status, prepregnancy body mass index (BMI, calculated as weight in kilograms divided by height in meters squared), maternal race and ethnicity (as a proxy for exposure to structural racism), parity, depressive symptoms (PHQ-9 [Patient Health Questionnaire-9]), and pregnancy health was collected from the EHR. Maternal education level was obtained from birth certificate data. Address at pregnancy onset was used to obtain median household income and unemployment rate of the Census tract of residence.

SAS 9.4 was used for all analyses. Overall, 32.4% of the observations had missing data for at least one covariate, ranging from 0.3% for prepregnancy BMI to 7.7% for maternal education level; 0.3% were missing gestational weight gain, and 19.7% were missing postpartum weight (Table 1). Multiple imputation was used to reduce bias from missing data for covariates (maternal education level, maternal smoking status, median household income of Census tract, unemployment rate of Census tract, prepregnancy BMI, maternal race and ethnicity, depressive symptoms, and parity) as well as the outcomes. Missing data patterns were evaluated and determined to be consistent with a missing-at-random pattern. Imputation was performed using the fully conditional specification and the discriminant function method that included all other covariates as the predictors for the variable being imputed. Relative efficiency and variance were examined to assess model performance, and both indicated adequate performance. Ten imputations were performed; the analytic models described below were run on each of the 10 imputed datasets. Results were then pooled using Rubin's rules.

**Table 1.** Descriptive Statistics for 935 Pregnant Individuals Referred to Wellness Coaching in Pregnancy, Stratified by Participation

	Participated in at Least 1 Session (n=525)	Did Not Participate in Any Sessions (n=410)
Sociodemographic characteristics		
Maternal age (y)	32.3±5.0	30.5±5.2
Household income of Census tract ($)	103,021±43,442	97,822±44,011
Missing	10 (1.9)	13 (3.2)
Maternal race and ethnicity		
Asian or Pacific Islander	129 (24.6)	76 (18.5)
Black	62 (11.8)	55 (13.4)
Hispanic	143 (27.2)	140 (34.1)
Non-Hispanic White	160 (30.5)	112 (27.3)
None of the above	17 (3.2)	16 (3.9)
Unknown or missing	14 (2.7)	11 (2.7)
Medicaid insurance	42 (8.0)	59 (14.4)
Maternal education level		
12th grade or less	68 (13.0)	84 (20.5)
Some college or technical school	136 (25.9)	126 (30.7)
College degree (BA or BS) or higher	281 (53.5)	168 (41.0)
Missing	40 (7.6)	32 (7.8)
Unemployment rate (% of Census tract)	3.4±2.1	3.6±2.2
Missing	7 (1.3)	11 (2.7)
Pregnancy health		
Prepregnancy BMI category		
Underweight	6 (1.1)	4 (1.0)
Normal weight	108 (20.6)	72 (17.6)
Overweight	159 (30.3)	114 (27.8)
Obesity	252 (48.0)	217 (52.9)
Missing	0 (0.0)	3 (0.7)
Parity		
Nulliparous	271 (51.6)	200 (48.8)
Multiparous	217 (41.3)	181 (44.2)
Missing	37 (7.1)	29 (7.1)
Smoking during early pregnancy		
Yes	15 (2.9)	19 (4.6)
No	489 (93.1)	364 (88.8)
Missing	21 (4.0)	21 (6.6)
PHQ-9 score	3.0 (1.0–6.0)	3.0 (1.0–6.0)
Missing	31 (5.9)	17 (4.1)
Gestational diabetes	64 (12.2)	43 (10.5)
HDP	171 (32.6)	146 (35.6)
Preterm birth	43 (8.2)	38 (9.3)
Weight outcomes		
Gestational weight gain adequacy ratio category		
Below IOM recommendations	109 (20.8)	75 (18.3)
Within IOM recommendations	73 (13.9)	60 (14.6)
Above IOM Recommendations	343 (65.3)	272 (66.3)
Missing	0 (0)	3 (0.7)
Postpartum weight retention (kg)	4.7±8.1	5.1±8.5
Missing	91 (17.3)	95 (23.2)
No. of months with postpartum weight available	2 (1–3)	2 (1–3)
Wellness Coaching in Pregnancy referral		
No. of referrals	1 (1–1)	1 (1–1)
Gestational age at referral (wk)	16.1 (10.6–24.3)	16.4 (10.3–24.6)
Wellness Coaching in Pregnancy participation		
No. of completed sessions	2 (1–2)	—
Trimester of 1st completed session		
1st	185 (35.2)	—
2nd	245 (46.7)	—
3rd	95 (18.1)	—

BA, Bachelor of Arts; BS, Bachelor of Science; BMI, body mass index; PHQ-9, Patient Health Questionnaire-9; HDP, hypertensive disorders of pregnancy; IOM, Institute of Medicine.

Data are mean±SD, n (%), or median (interquartile range).

Inverse probability of treatment weighting was used to reduce confounding bias (because participants were not randomized to Wellness Coaching in Pregnancy in this observational study) by balancing characteristics between individuals who participated in Wellness Coaching in Pregnancy and those who did not. The inverse probability of treatment weights was calculated using a propensity score model. The distributions of covariates were compared before and after weighting by the stabilized inverse probability of treatment weights. All candidate variables (a priori–identified potential confounders and precision variables: maternal age, maternal education level, maternal smoking status, median household income of Census tract, unemployment rate of Census tract, prepregnancy BMI, maternal race and ethnicity, depressive symptoms, and parity) were retained in the final model as satisfactory balance was achieved using this model. The standardized differences between groups for each of the variables fell well below the threshold of 0.1 (determined a priori) after weighting.^24^

We stratified descriptive statistics by participation in Wellness Coaching in Pregnancy. Continuous variables were summarized using mean and standard deviation or median and interquartile range. Categorical variables were summarized using frequency and percentage.

Multinomial logistic regression, weighted by inverse probability of treatment weights, was used to estimate associations of Wellness Coaching in Pregnancy participation with odds of gestational weight gain above and below IOM guidelines. A repeated measures mixed-effects linear model, weighted by inverse probability of treatment weights, was used to estimate associations of Wellness Coaching in Pregnancy participation with mean difference in postpartum weight retention (kg). Initial models included a multiplicative interaction term between Wellness Coaching in Pregnancy participation and postpartum month of weight measurement to test whether associations of Wellness Coaching in Pregnancy participation with postpartum weight varied by postpartum month. We did not find significant (*P*>.05) interaction and present results from a model without interaction.

To address the possibility that results by trimester of first completed Wellness Coaching in Pregnancy session may be driven by total number of sessions completed (because participants who initiated Wellness Coaching in Pregnancy in the first trimester would have more time to complete multiple Wellness Coaching in Pregnancy sessions compared with those who initiated Wellness Coaching in Pregnancy later in pregnancy), rather than timing of participation, we additionally stratified analyses for number of sessions completed by trimester of first Wellness Coaching in Pregnancy session. We also adjusted for gestational diabetes diagnosis to account for the potential influence of associated diet therapy on maternal weight outcomes.

## RESULTS

**Fig. 1.**
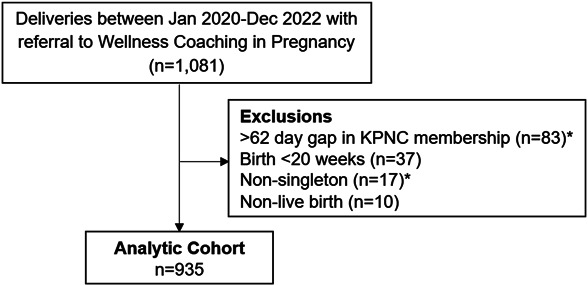
Flow diagram for study inclusion. *One patient was excluded for two reasons (more than 62-day gap in membership and nonsingleton birth). KPNC, Kaiser Permanente Northern California.

From 2020 to 2022, 935 pregnant individuals had at least one referral to Wellness Coaching in Pregnancy in Pregnancy (Fig. 1). The median number of referrals during pregnancy was one, at a median gestational age of 16 weeks. About half of those referred had prepregnancy obesity and were nulliparous. Among the 935 referred pregnant individuals, 525 (56.1%) participated in at least one Wellness Coaching in Pregnancy session after a referral, with a median of two completed sessions. Comparing individuals who did not participate in Wellness Coaching in Pregnancy after referral with those who participated in at least one session (Table 1), those who participated in Wellness Coaching in Pregnancy were slightly older, more likely to be Asian or Pacific Islander, more likely to have a college degree or higher, and were less likely to be Hispanic, have Medicaid insurance, or have prepregnancy obesity.

*Badon. Health coaching and maternal weight outcomes. O&G Open 2025*.

Participation in Wellness Coaching in Pregnancy was not associated with the gestational weight gain category (Table 2) or postpartum weight retention (Table 3) overall or when stratified by number of sessions completed. However, we observed associations by trimester of first completed Wellness Coaching in Pregnancy session (Tables 2 and 3). Completing the first session of Wellness Coaching in Pregnancy in the first trimester was associated with 40% lower odds of gestational weight gain above IOM recommendations (odds ratio 0.60; 95% CI, 0.39–0.94) and 2.2 kg lower mean postpartum weight retention up to 14 months (95% CI, −3.7 to −0.7). Completing the first session in the third trimester was associated with higher odds of gestational weight gain above IOM recommendations (adjusted odds ratio 1.24, 95% CI, 1.08–4.66), but not with postpartum weight retention (adjusted mean difference 0.9 kg; 95% CI, −1.0 to 2.9).

**Table 2.** Association of Participation in Wellness Coaching in Pregnancy With Institute of Medicine Gestational Weight Gain Categories

	Below IOM Recommendations		Within IOM Recommendations (n)*	Above IOM Recommendations	
	n	OR (95% CI)		n	OR (95% CI)
Overall					
No participation	56	Ref	65	286	Ref
Any participation	71	0.86 (0.54–1.38)	99	355	0.87 (0.62–1.23)
No. of Wellness Coaching in Pregnancy sessions completed					
No participation	56	Ref	65	286	Ref
1	36	0.91 (0.52–1.59)	48	149	0.77 (0.50–1.17)
2	17	0.69 (0.35–1.37)	30	128	1.03 (0.64–1.66)
3 or more	18	1.01 (0.49–2.09)	21	78	0.87 (0.50–1.52)
Trimester of 1st completed Wellness Coaching in Pregnancy session					
No participation	56	Ref	65	286	Ref
1st	38	1.11 (0.63–1.95)	42	105	0.60 (0.39–0.94)
2nd	28	0.70 (0.39–1.26)	49	168	0.84 (0.55–1.27)
3rd	5	0.59 (0.18–1.92)	8	82	2.24 (1.08–4.66)

IOM, Institute of Medicine; OR, odds ratio; Ref, referent.

* Reference group.

**Table 3.** Association of Participation in Wellness Coaching in Pregnancy With Postpartum Weight Retention Up to 14 Months

	n	Mean Difference (kg) (95% CI)
Overall		
No participation	313	Ref
Any participation	434	−0.3 (−1.4 to 0.9)
No. of Wellness Coaching in Pregnancy sessions completed		
No participation	313	Ref
1	193	−0.1 (−1.6 to 1.3)
2	139	0.2 (−1.4 to 1.8)
3 or more	102	−1.1 (−2.9 to 0.6)
Trimester of 1st completed Wellness Coaching in Pregnancy session		
No participation	313	Ref
1st	150	−2.2 (−3.7 to −0.7)
2nd	208	0.7 (−0.7 to 2.0)
3rd	76	0.9 (−1.0 to 2.9)

Ref, referent.

In sensitivity analyses stratifying analyses for number of sessions completed by trimester of first Wellness Coaching in Pregnancy session, results were generally similar to analyses by trimester of Wellness Coaching in Pregnancy initiation that did not consider number of sessions completed (Appendices 2 and 3, available online at http://links.lww.com/AOG/E358). Importantly, among individuals who initiated Wellness Coaching in Pregnancy in the first trimester, completing only one session of Wellness Coaching in Pregnancy was associated with lower odds of gestational weight gain above IOM recommendations (adjusted odds ratio 0.47; 95% CI, 0.25–0.88) and 3.1 kg lower postpartum weight retention (95% CI, −5.4 to −0.9). Results after additional adjustment for gestational diabetes were similar to main results (Appendices 4 and 5, available online at http://links.lww.com/AOG/E358).

## DISCUSSION

In this study of pregnant individuals referred to a telephone-based health coaching program within an integrated health care delivery system, Wellness Coaching in Pregnancy participation overall was not associated with gestational weight gain or postpartum weight retention. When Wellness Coaching in Pregnancy was initiated in the first trimester, there was an association with lower odds of gestational weight gain above IOM recommendations and lower postpartum weight retention. There were no beneficial associations with weight outcomes for initiation of health coaching in the second or third trimester.

Our findings suggest that early pregnancy is a critical period for intervention to prevent excessive gestational weight gain. A previous study reported that individuals with obesity who exceeded gestational weight gain recommendations started to accelerate weight gain between 12 and 14 weeks of gestation and accumulated most of their total gestational weight gain in the second trimester.^25^ Health coaching in the first trimester may allow behavior change to take place before this period of accelerated gestational weight gain. Our results align with a recent review of lifestyle interventions delivered within health care systems that targeted gestational weight gain, which found that all effective programs for individuals with overweight or obesity were initiated in the first half of pregnancy.^26^ Similarly, a study of a health system–based telephone coaching program in Australia reported a 36% lower absolute risk of gestational weight gain above IOM recommendations among individuals who began coaching by 16 weeks of gestation, though benefits were limited to those with prepregnancy obesity.^20^ Our study extends these findings to a more diverse population across a broader range of BMI categories.

Timing of gestational weight gain also likely explains why we observed that initiating Wellness Coaching in Pregnancy in the third trimester was associated with higher odds of gestational weight gain above recommendations. This may reflect reverse causality: individuals who initiated Wellness Coaching in Pregnancy later in pregnancy may have already gained more than recommended, prompting referral or personal motivation to initiate Wellness Coaching in Pregnancy. Interventions in late pregnancy may have limited influence on gestational weight gain.

We also observed beneficial associations of initiating health coaching in early pregnancy for postpartum weight retention. These results are consistent with findings from a randomized trial of telephone-based support delivered by research staff beginning at 13 weeks of gestation that also reported lower postpartum weight retention at 12 months postpartum.^27^ Because excessive gestational weight gain is a well-established risk factor for postpartum weight retention,^7–9^ the observed association of early initiation of Wellness Coaching in Pregnancy with lower postpartum weight retention may be mediated through healthier gestational weight gain. Initiation of health coaching in early pregnancy also may support habit formation and behavior maintenance. Among nonpregnant adults, interventions that last 24 weeks or more have been associated with sustained health behavior change.^28^ The individualized nature of health coaching may have further contributed to sustained behavior change by supporting goal setting, problem-solving, and personalized strategies—features known to enhance nutrition counseling effectiveness in nonpregnant adults.^29^

Our findings support referral to individualized telephone-based nutrition coaching or counseling in the first trimester to reduce the risk of excessive gestational weight gain and postpartum weight retention. Depending on program capacity, clinicians may consider prioritizing individuals at higher risk, including those with a prepregnancy BMI of 25 or higher, insulin resistance or diabetes, polycystic ovary syndrome, or depression.^30^ Further work is needed to understand how best to promote referral and participation in health coaching programs during pregnancy.

Strengths of this study include its pragmatic focus, evaluating an established health coaching program within a large health care delivery system and using routinely collected EHR data to identify participation and weight outcomes; consideration of both pregnancy and postpartum weight outcomes; exploration of dose response and timing; use of gestational weight gain adequacy ratio to categorize gestational weight gain allowed classification across all gestational ages of birth, including preterm births; use of propensity score weighting to account for the nonrandomized nature of participation in health coaching and to adjust for important confounders; and use of multiple imputation to handle missing data.

There are also several limitations that should be considered when interpreting our results. Referral to Wellness Coaching in Pregnancy typically followed clinician counseling and patient interest in the program, meaning our study population may have been more motivated than the general pregnant population. By limiting our study population to individuals who were referred to health coaching, we have attempted to minimize differences in motivation or readiness for change between those who participated in Wellness Coaching in Pregnancy and those who did not, but individuals who initiated health coaching in early pregnancy may have differed in unmeasured ways that our propensity score weighting may not have fully accounted for. Finally, all participants were members of a large integrated health care delivery system, and results may not be generalizable to other settings.
